# Primary hemiarthroplasty for treatment of unstable pertrochanteric femoral fractures (AO/OTA Type 31 A2.3) in elderly osteoporotic patients

**DOI:** 10.1051/sicotj/2017013

**Published:** 2017-04-07

**Authors:** Skender Ukaj, Bahri Gjyshinca, Vlora Podvorica, Fatime Ukaj, Granit Molliqaj, Arben Boshnjaku, Axel Gamulin

**Affiliations:** 1 Department of Orthopedics and Osteo-Muscular Trauma, University Clinical Center of Kosovo 10000 Pristina Kosovo; 2 Neurosurgical Department, University Hospital of Geneva 1200 Geneva Switzerland; 3 Faculty of Medicine, University of Gjakova Fehmi Agani 50000 Gjakove Kosovo; 4 Division of Orthopaedic and Trauma Surgery, Department of Surgery, University Hospitals of Geneva 1200 Geneva Switzerland

**Keywords:** Unstable pertrochanteric fracture, Primary hemiarthroplasty, Osteoporotic patients, Elderly

## Abstract

*Introduction*: The aim of this study was to prospectively analyze the role of primary hemiarthroplasty in unstable osteoporotic pertrochanteric fractures (AO/OTA Type 31 A2.3), with emphasis given to postoperative Functional Independent Measure (FIM) and Harris Hip Score (HHS).

*Methods*: Fifty-six consecutive patients (average age 78.25 ± 5.45), out of which 24 males (79.29 ± 4.99) and 32 females (77.47 ± 5.72), with unstable pertrochanteric femoral fractures, operated with primary hemiarthroplasty procedure from 2012 to 2014 were included in this prospective study with a follow-up of two years. Primary outcomes were FIM and HHS. Secondary outcomes included duration of surgery, estimated intraoperative blood loss, time to first postoperative full weight-bearing, time to walking ability with and without crutches, average hospital stay, postoperative complications, and mortality.

*Results*: The FIM score at 3 months was 85.9 ± 5.7. HHS at two years was excellent for 41 patients (73, 2%), good for eight (14.3%), fair for four (7.1%), and poor for three (5.4%). The mean duration of surgery was 62.6 min, estimated intraoperative blood loss 175.5 mL, time to first postoperative full weight-bearing 2.2 ± 0.4 days, ability to walk with crutches 6.3 ± 1.8 days and without crutches 44.2 ± 12.7 days, and the average hospital stay was 9.6 ± 2.7 days.

*Conclusion*: This study highlighted good clinical postoperative outcome scores for primary hemiarthroplasty for the treatment of unstable pertrochanteric femoral fractures in elderly osteoporotic patients. This procedure seems to be secure and effective, and offers a good quality of life in terms of FIM and HHS.

**Abbreviations**
HHC: Harris Hip Score
FIM: Functional Independence Measure
UCCK: University Clinical Center of Kosovo
CEPI: Committee of Ethical and Professional Issues

## Introduction

More than 280 000 hip fractures occur in the United States each year, and this incidence is expected to double by 2050 [1]. Pertrochanteric fractures represent 45% of all hip fractures and usually occur as a result of a minor trauma on preexisting osteoporosis in an elderly patient [2, 3]. Among pertrochanteric fractures, 35–40% are unstable (three or four part fracture, AO/OTA Type 31 A2.3; 15% of all hip fractures) [2, 4]. Optimal treatment and management of these unstable pertrochanteric femoral fractures in elderly osteoporotic patients remains a challenge in current orthopedic trauma practice. Internal fixation may be associated with nonanatomic reduction of fracture fragments, long bed rest periods, prolonged protected weight-bearing, bone fragment necrosis, and secondary loss of reduction due to unstable fixation in poor quality bone [5–8]. Moreover, these fractures are associated with substantial morbidity and mortality; about 30% of elderly patients die within one year of fracture [2, 9].

The aim of this study was to prospectively analyze the role of primary hemiarthroplasty in unstable osteoporotic pertrochanteric fractures (AO/OTA Type 31 A2.3), with emphasis given to postoperative full weight-bearing and return to normal quality of life.

## Materials and methods

Following institutional review board’s approval (central archive No. 1035), 56 consecutive patients aged 65 or over with an unstable pertrochanteric femoral fracture (AO/OTA Type 31 A2.3) were prospectively included in this study between January 2012 and January 2014.

Inclusion criteria were: (1) AO/OTA Type 31 A2.3 fracture; (2) patient aged 65 or over; (3) informed consent obtained. Exclusion criteria included: (1) patients with pathological fractures; (2) patients with any type of neurological disorder that could affect (directly or indirectly) bone density or future recuperation (such as paresis or hemiparesis, multiple sclerosis, Parkinson’s disease, etc.); (3) patients with a previous contralateral pertrochanteric fracture; (4) patients with preexisting coxarthrosis in the same hip. Patient demographic factors that were included in the analysis were age, gender, fracture type, mechanism of injury, and preinjury mobility level. There was no dual-energy X-ray absorptiometry (DEXA) examination to determine if osteopenia or osteoporosis was present at the time of the injury, leaving evaluations to be done only by X-ray scans.

Primary outcomes were postoperative Functional Independent Measure (FIM) and Harris Hip Score (HHS). These scores were obtained at three, six, 12, and 24 months follow-up examinations together with antero-posterior (AP) and axial plain radiographs. Secondary outcomes included duration of surgery, estimated intraoperative blood loss, time to first postoperative full weight-bearing, time to walking ability with and without crutches, average hospital stay, postoperative complications (infections, leg length discrepancy, prosthetic dislocation, sciatic nerve palsy, deep venous thrombosis (DVT), and mortality).

### Surgical technique

All patients were operated under spinal anesthesia, within 2.3 ± 0.7 days (range 1–3) from their admission. AP and axial plain radiographs were used for appropriate preoperative planning. Patients were operated on in lateral positioning, and a posterior approach was used to expose the proximal femur, the capsule, and the acetabulum.

**Figure 1.**
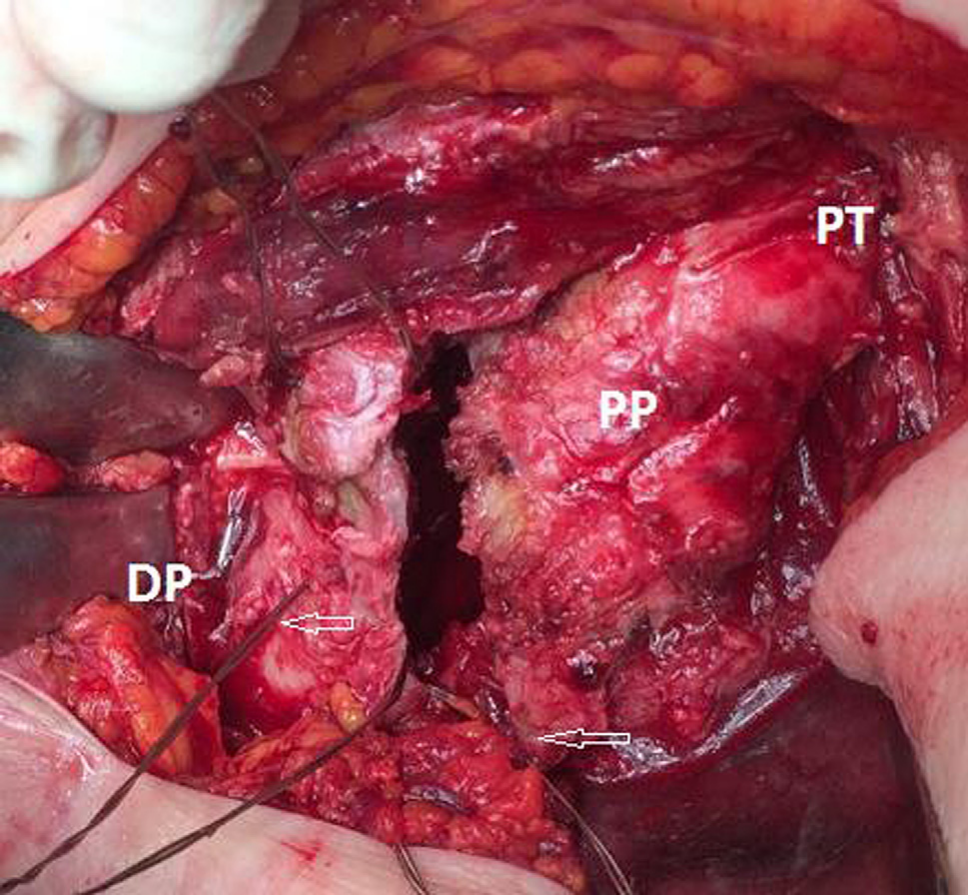
Determination of femur length, fixation of greater trochanter by tension band wires.

The joint capsule was opened using a T-shaped capsulotomy and the femoral head was extracted and measured (Figure 1). The femoral neck was cut following the preoperative planning measurement. Temporary reduction and fixation of the greater and lesser trochanter were performed to determine femoral length and antetorsion. Femoral canal preparation was then undertaken, using progressive rasps to achieve a good purchase of the trial implant into the shaft. The range of motion and joint stability were checked with the trial implants in place.

**Figure 2.**
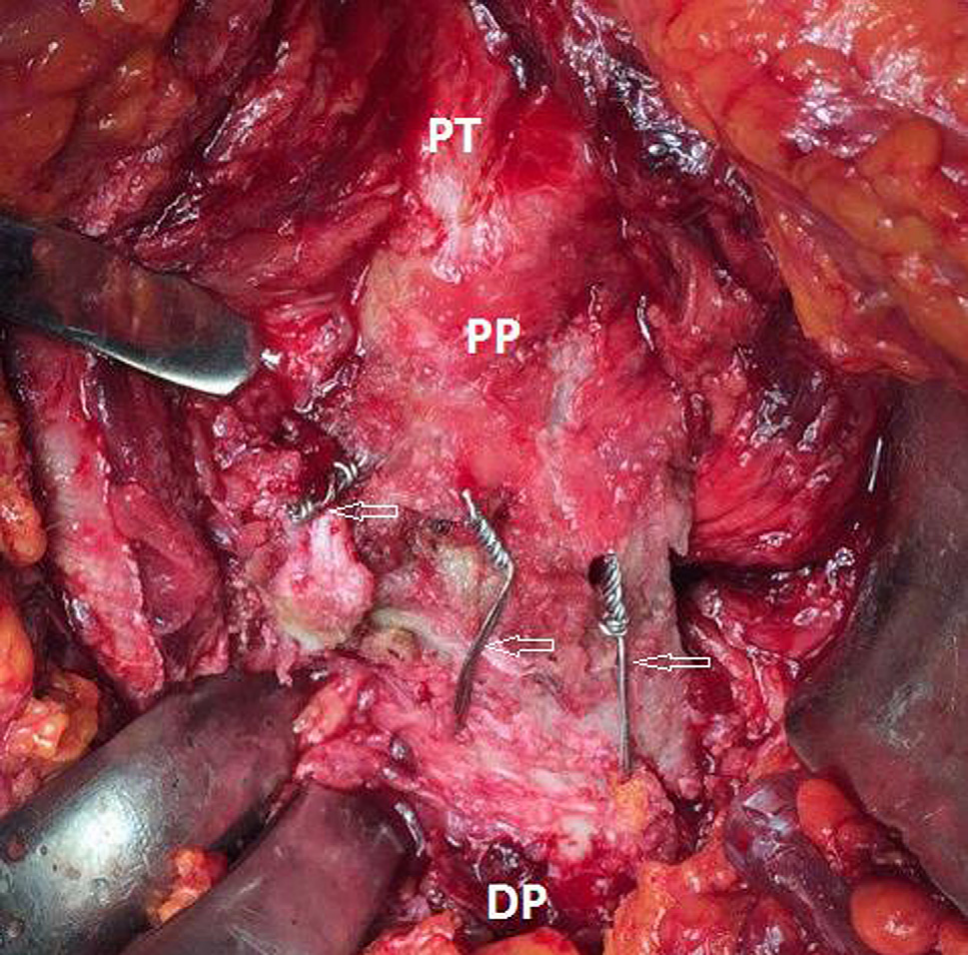
Anatomical positioning and fixation of the lesser trochanter.

**Figure 3.**
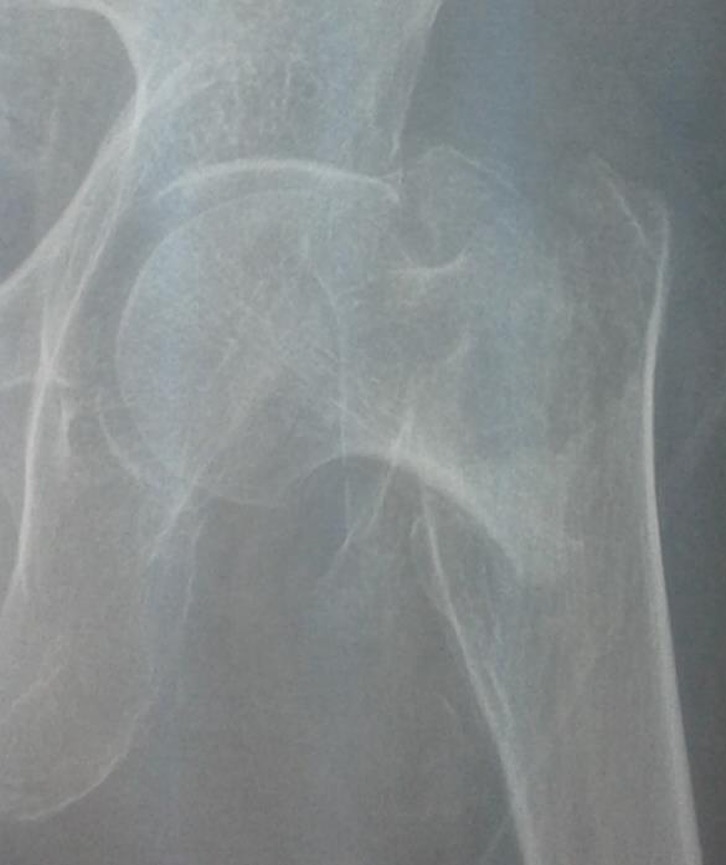
Pre operation X-ray.

**Figure 4.**
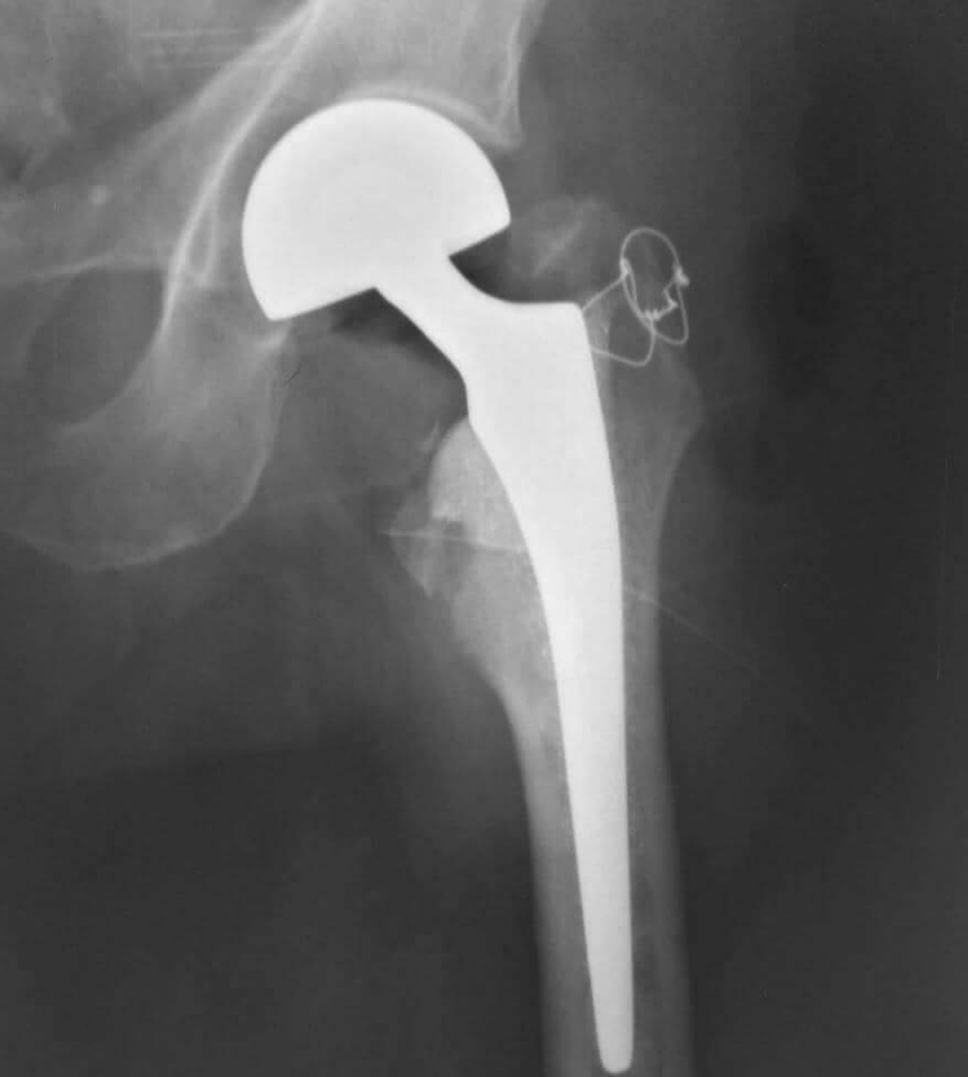
Post operation X-ray.

Definitive greater trochanter fixation was done by tension band wires inserted in holes drilled in the proximal and distal fragments. The lesser trochanter was also reduced and fixed using tension band wiring (Figure 2). After fragment fixation, cemented femoral stems were used in all the cases because of poor bone quality. Low viscosity cement was used due to better penetration through drilled holes. For patients under 85 years old bipolar hemi-prostheses were used, and for patients over 85 years old Austin-Moore hemi-prostheses were used. The range of motion and stability were checked again. The capsule was repaired, the short external rotators reattached, and the wound closed over a suction drain. Preoperative (Figure 3) and postoperative (Figure 4) radiographs were obtained. In order to prevent the deep venous thrombosis (DVT), we used fractionized heparin and bilateral elastic stockings. Patients were allowed full weight-bearing ambulation on the first postoperative day.

## Statistical analysis

Statistical analysis was performed using SPSS program for statistical analysis, version 12.0 for windows, and statistical significance set at *p* < 0.05.

Descriptive statistics was generated for all the study variables, including the mean average for continuous variables and relative frequencies for categorical variables. Differences between groups with continual data were performed using unpaired *t* test with Welch’s correction (to compare two groups).

## Results

Prospectively collected clinical and radiological data from 56 consecutive patients operated for unstable osteoporotic pertrochanteric fractures (AO/OTA Type 31 A2.3) with primary hemiarthroplasty procedure were assessed. The collective included 32 women and 24 men with a mean age of 78.3 ± 5.5 years (range 70–92). The average age of males was 79.29 ± 4.99 years and that of females 77.47 ± 5.72 years. Forty-five patients were injured after falls from a standing height, four during road traffic accidents, and seven while biking. There were no bilateral fractures, no patient already had a contralateral proximal femoral fracture, and there were no associated injuries in any patient. The surgery was performed within 2.3 ± 0.7 days (range 1–3 days) from the admission (Table 1).

**Table 1.** Participants and operations characteristics.

	Male (*n* = 24)	Female (*n* = 32)	*P* value	Total (*n* = 56)
Gender distribution	42.9%	57.1%		100%
Age	79.3 ± 5.0	77.5 ± 5.7	*p* < 0.05	78.3 ± 5.5
Operation duration (min)	62.5 ± 5.7	62.7 ± 6.4	*p* > 0.05	62.6 ± 6.0
Blood loss intraop (mL)	171.5 ± 38.2	178.6 ± 26.3	*p* < 0.05	175.5 ± 31.8
Surgery after admission (days)	2.3 ± 0.7	2.3 ± 0.7	*p* > 0.05	2.3 ± 0.7
Hospitalization (days)	9.5 ± 2.7	9.6 ± 2.8	*p* > 0.05	9.6 ± 2.7

The FIM score at three months was 85.9 ± 5.7. The HHS at two years was excellent for 41 patients (73.2%), good for eight (14.3%), fair for four (7.1%), and poor for three (5.4%).

The mean duration of surgery was 62.6 ± 6.0 min (range 55–70), mean estimated intraoperative blood loss was 175.5 ± 31.8 mL (range 115–215), mean time to first postoperative full weight-bearing was 2.3 ± 0.4 days (range 2–3), mean time to being able to walk was 29.8 ± 3.1 days (range 28–35) with crutches and 44.3 ± 12.7 days (range 28–63) without crutches, and average hospital stay was 9.6 ± 2.7 days (range 4–14). Postoperative complications were reported as follows: one case of superficial infection which was treated with a course of antibiotics including third generation of cephalosporins and gentamicin; four patients with shortening of their lower limb of less than 2 cm and three patients with a lengthening of 1.5 cm. No case that lost the walking ability permanently was recorded in our study, yet, three patients continued using one crutch even after 24 months follow-up. There was no postoperative prosthetic dislocation during the follow-up period, and there was no postoperative sciatic nerve palsy nor deep venous thrombosis. Six patients died during the two years follow-up period.

## Discussion

Cephalomedullary nail fixation has been reported as the treatment of choice for unstable multifragmentary pertrochanteric fractures [10]. Sliding hip screw devices have also been used by some authors in combination with the trochanter stabilizing plate, trochanteric screws, or tension band wires [11, 12]. However, after osteosynthesis of an unstable pertrochanteric fracture, early active mobilization and full weight-bearing are delayed in order to prevent secondary displacement of fracture fragments [13, 14]. Since most elderly patients have difficulties in respecting partial weight-bearing recommendations, early active mobilization and walking rehabilitation may be postponed for many weeks, until first signs of bony consolidation appear on control radiographs [15]. Fracture healing in the geriatric patient should be normally completed within three to five months [15]. Since early postoperative mobilization and full weight-bearing is a major factor improving quality of life and reducing postoperative complications (pulmonary complications, venous thrombosis, pressure sores, generalized deconditioning) and mean length of hospital stay [16], any delay in walking rehabilitation may be detrimental to the patients and should be avoided.

Prosthetic replacement in these cases may play an important role, by allowing full weight-bearing. However, the indications for such a procedure are not yet well defined: one major indication might be a comminuted and unstable pertrochanteric fracture in an elderly and debilitated patient with osteopenic or osteoporotic bone [2, 3, 5, 17, 18].

Dual mobility cup (DMC) has also been shown as an effective solution for management of high risk cases undergoing total hip replacement (THR), in order to reduce the incidence of postoperative instability [19]. This technique shows that there is no impingement between the neck of prosthesis and acetabular shell at extremes of all movements [19]. Nevertheless, the intraprosthetic dislocation (IPD) is peculiar to it [20], even though lower than in many other techniques.

Probably one of the best proved techniques so far for treating subtrochanteric and extra-capsular trochanteric fractures (ECTF) is the transtrochanteric approach with coronal osteotomy of the great trochanter. This technique is performed by internal fixation with proximal locking nail or sliding hip screws [21], but which gets tricky when dealing with patients with severe osteoporosis.

Hemiarthroplasty has been used for unstable intertrochanteric fractures since 1971 [22]. There are multiple studies showing good results using this technique. Stern and Goldstein used the Leinbach prosthesis for the primary treatment of 22 AO/OTA Type 31A2.3 pertrochanteric fractures and found early ambulation and early return to the pre-fracture status as a definite advantage [22]. Primary arthroplasty provides adequate fixation and allows early mobilization and weight-bearing thus decreasing postoperative complications. Grimsrud et al*.* showed that AO/OTA Type 31A2.3 fractures can be safely treated with a standard femoral stem and cerclage wiring of both trochanters: the technique allows safe and early weight-bearing on the injured hip and has a low rate of complications [4]. Hemi-prosthetic replacement was also recommended by other authors in the treatment of AO/OTA Type 31A2.3 fractures in order to avoid fracture instability and to allow early postoperative weight-bearing [18, 23].

A prospective randomized study comparing compression hip screws to Vandeputte hemi-prosthesis in these fractures suggested that primary prosthetic replacement might have fewer early complications and satisfactory functional results [18]. Other studies have also shown a higher rate of complications in patients treated with osteosynthesis when compared to arthroplasty, leading to more application of arthroplasty techniques [5].

Some studies showed a slightly better functional outcome with arthroplasty, yet with no statistically significant difference (*p* > 0.05) [2]. The postoperative dislocation rate in total hip arthroplasty after intertrochanteric fractures was reported to be as high as 40%, but was much lower in hemiarthroplasty [24]. Kayali et al. in a comparative study of hemiarthroplasty versus internal fixation reached the conclusion that clinical results of both groups were similar, but hemiarthroplasy patients were allowed full weight-bearing earlier [25]. In the comparison done by Haentjens & Lamraski, the incidence of pneumonia and pressure sores was significantly reduced in arthroplasty patients [26]. Surgeons may worry about blood loss amount during arthroplasty, but Broos et al. in their retrospective study showed that operative time, blood loss, and mortality rate were comparable between arthroplasty and internal fixation [23, 27].

Therefore, based on the above-mentioned facts, hemiarthroplasty presents the ideal technique for treating unstable intertrochanteric fractures. But, when comparing Leinbach prosthesis where the implantation of endoprosthesis is needed firstly before the fixation of both trochanters, to standard femoral stem (that we used) where the fixation of both trochanters is done before the cemented femoral stem (in all cases) we are convinced that the probability of intra- and postoperative complications is lower (such as prevention of ante- and retroversion, as well as the length of leg). Adding here the fact that the possibility of having an approach in the particular (modular) prosthesis depends on its availability and economical costs, this presents the appropriate technique for treating these cases. Another important factor that emphasizes the importance of this specific technique (that was used in our study) is the low mortality rate. From six patients who died during the follow-up period, none of them died due to intrahospital stay, but all the cases were because of other associated complications.

This study has several limitations, such as: (1) no osteodensitometry scan was performed in our patients in order to assess bone density, and patients were deemed osteopenic on the basis of standard radiographs; (2) the Austin-Moor hemi-prosthesis that was used in patients over 85 years old was a bit old fashioned (comparing to bipolar hemi-prosthesis that were used in those under 85 years old), but our decision was mainly influenced by economic reasons and the low cost that Austin-Moor hemi-prosthesis presents (comparing to their counterpart); (3) there was no control group of patients operated on with an osteosynthesis technique to make a proper comparison.

## Conclusion

As the general population is getting older while staying active, faster and more complete rehabilitation as well as decreased complication rate must be achieved for these patients. In the present study, primary hemiarthroplasty for the treatment of unstable pertrochanteric femoral fractures (AO/OTA type 31 A2.3) in elderly osteoporotic patients seems to be a secure and effective procedure, while showing an earlier ability to bear full body weight, a faster recovery rate, and an improved quality of life. This approach offered a suitable improved quality of life in terms of FIM and HHS. Early mobilization is advantageous in preventing pulmonary complications, venous thrombosis, pressure sores, and generalized deconditioning. Future supportive studies are required to prove our hypothesis in terms of confidence and reliability.

## Conflict of interest

All the authors declare no conflict of interest.
